# Effects of Graphene Oxide on the Structure and Properties of Regenerated Wool Keratin Films

**DOI:** 10.3390/polym10121318

**Published:** 2018-11-28

**Authors:** Bo Li, Jinbo Yao, Jiarong Niu, Jianyong Liu, Le Wang, Mao Feng, Yanli Sun

**Affiliations:** 1 School of Textiles, Tianjin Polytechnic University, Tianjin 300387, China; 1988li01bo22@163.com (B.L.); jianyong1964@126.com (J.L.); wangletianjin@163.com (L.W.); 52x@msn.com (M.F.); yls198959@163.com (Y.S.); 2 School of Chemistry and Chemical Engineering, Wuhan Textile University, Wuhan 430200, China; 3 Hubei Key Laboratory of Biomass and Eco-Dyeing & Finishing, Wuhan Textile University, Wuhan 430200, China

**Keywords:** wool keratin, graphene oxide, crystallinity, mechanical properties

## Abstract

Much research has focused on improvement of the structural and mechanical properties of regenerated keratin materials by physical or chemical methods in recent years. In this research, regenerated keratin materials were modified with graphene oxide (GO). The properties of modified keratin films and the mechanism of interaction between GO and keratin macromolecules were studied. The SEM and XRD test results showed that the orientation of keratin macromolecules could be effectively improved by GO, which favored improvement of the keratin material’s crystallinity and made the films more uniform and compact. The thermal stability and mechanical properties of GO-modified keratin films were also improved significantly. At the same time, the reaction mechanism between keratin and GO materials was analyzed by sodium dodecyl sulfate-polyacrylamide gel electrophoresis (SDS-PAGE), FT-IR, and Raman spectroscopy. It was shown that there was no chemical reaction between GO and keratin molecules, and the interaction between them was mainly via hydrogen bonding and van der Waals forces.

## 1. Introduction

Wool fiber is one of the earliest fibers used by human beings and is often used to produce high-grade products. Wool fibers contain a large amount of keratin (approximately 95%) [1,2], which has many advantages, such as high molecular weight and good biocompatibility and degradability [3,4,5]. In recent years, many researchers have attempted to obtain new biopolymer materials with excellent properties through regenerated keratin. However, effectively improving regenerated keratin materials’ mechanical properties is difficult and is an important topic in this research field [6,7,8].

The outstanding physical properties of wool fibers are derived from their complex chemical groups on keratin macromolecules and their fine arrangement [9,10,11]. In the dissolving process of wool fibers, the secondary structure between keratin molecules is destroyed, and these broken molecular chains are disordered in the solution. Finally, the macromolecules form a three-dimensional network that is cross-linked in regenerated materials. This is the main reason that keratin materials are brittle and hard. At present, researchers have two main methods to overcome mechanical defects. One method is to blend keratin with other kinds of polymers such that the deficiency of keratin materials can be compensated for by the excellent mechanical properties of the added polymers. However, the addition of polymers is often excessive and the final products are not considered pure keratin materials [12,13,14]. The other method is that the keratin is physically or chemically modified. In this way, the mechanical properties are improved through sealing parts of the side groups on the macromolecular chains, thus increasing the average molecular weight of the keratin during regeneration, or adjusting the arrangement of the molecules. This method has the advantage of being able to prepare pure regenerated keratin material that retains many of the excellent properties of keratin [15,16,17].

**Figure 1:**
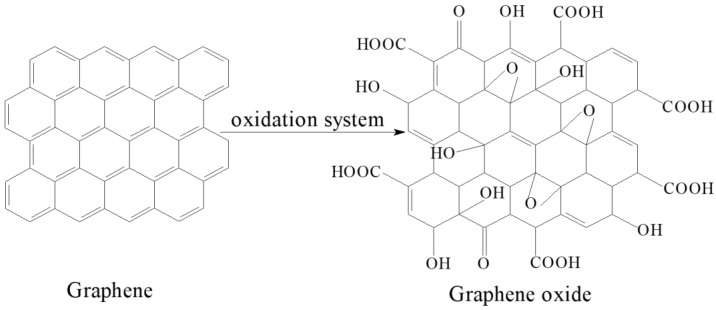
Structure schematic of graphene and graphene oxide.

The selection of a suitable modifying reagent is crucial in keratin modification. Graphene and graphene oxide have become the focus of research in recent years [18,19,20,21]. Graphene is a non-polar material that it is not easily soluble in water. However, graphene oxide has richer physicochemical properties, including good water solubility. This difference is due to the presence of oxygen-containing polar groups and planar edge oxygen-containing polar groups on both sides of graphene oxide material’s carbon plane [22,23], the basic structure of the graphene oxide material is shown in Figure 1.

The surface of graphene oxide has a large number of oxygen-containing functional groups which can form hydrogen bonds or other chemical bonds with other materials, and thus graphene oxide is commonly used as a reinforcing matter in preparation of composite materials. Xu et al. reported that polyamide 6 (PA6) can reacted with the double bonds of a GO surface and PA6 molecules grafted onto the GO surface. Thus, modified GO can be uniformly dispersed in the matrix of PA6, which can then be prepared into fibers by the melt spinning method. The research showed that when the amount of GO was 0.10 wt %, the tensile breaking strength and elastic modulus of the composite fibers compared with PA6 fibers were increased by 2.1 times and 2.4 times, respectively [24]. Esparza et al. found that a kind of thermal plastic material could be produced when chicken feather was treated with sodium sulfite in a mixture of glycerol/propylene glycol and water at 150 °C. The mechanical properties of the plastic were significantly improved by adding 1 wt % graphene oxide. The research suggested that the interaction between protein and GO could be improved through increasing proportion of hydroxyl groups and interlayer space in graphene oxide [23].

At present, there are many studies on the application of graphene and graphene oxide to improve the mechanical properties of composite materials, but research on regenerated keratin materials modified by graphene oxide is rare. The structure and mechanical properties of keratin materials were improved by the modification process, which made them suitable for the preparation of regenerated protein fibers. In this study, graphene oxide was used to modify regenerated wool keratin. The mechanism of graphene oxide improvement of wool keratin’s properties was discussed based on Fourier transform infrared analysis (FT-IR), Raman spectroscopy, and X-ray diffraction (XRD). Meanwhile, the structure and properties of modified keratin films were examined by scanning electron microscopy (SEM), differential scanning calorimetry (DSC), thermogravimetric analysis (TG), and tensile strength testing.

## 2. Experimental

### 2.1. Materials

The cleaned wool fibers were supplied by Zhejiang Xinao Textile Inc.,Tongxiang, China. Lks-610 reagent (40%, pH = 8, Tianjin Lvyuan Tianmei Technology Co., Ltd., Tianjin, China) and formic acids solution (88%, Kemiou Chemical Reagent Co., Ltd., Tianjin, China) were used to dissolve wool fibers. *β*-mercaptoethanol was supplied by Tianjin Fengchuan Chemical Reagent Technologies Co., Ltd., Tianjin, China. Sodium dodecyl sulfate (SDS) and glycerol were obtained from Tianjin Guangfu Fine Chemical Research Institute, Tianjin, China. Coomassie brilliant blue and bromophenol blue were acquired from Nanjing Oddfoni Biological Technology Co., Ltd., Nanjing, China. Graphene oxide was purchased from Qingdao AoKe ShiMo Co. Ltd., Qingdao, China. All the reagents used in this research were of analytical grade. 

### 2.2. Preparation and Modification of Regenerated Keratin Film

#### 2.2.1. Preparation of Wool Keratin Solution

Wool keratin solution was prepared by dissolving wool fibers in reducing agent and formic acid solution. One gram of clean, dried wool fibers was cut into pieces and treated with 10 mL LKS-610 reagent at 80 °C for 60 min. After pre-treatment, the excess reagent in the fibers was filtered out and the fibers’ liquid ratio was 100%. The pre-treated fibers were immersed in 20 mL formic acid solution and dissolved for 5 h at 60 °C. Finally, the keratin solution was centrifuged and filtered to remove the deposits and impurities.

#### 2.2.2. Modification of Regenerated Keratin with Graphene Oxide

Graphene oxide powder was added into the wool keratin solution at the ratio of 1 wt % of keratin. The solution was stirred for 3 h at ambient temperature with a magnetic mixer, and then shocked for 4–5 h in an ultrasonic oscillator at 40 °C. Then the solution was defoamed for use.

#### 2.2.3. Preparation of Regenerated Keratin Films

Keratin solution was cast into a glass sink evenly and the liquid thickness was controlled to 1 mm. Then, it was dried at 40 °C for 24 h to form a film. The unmodified keratin film was named as KF and the modified one as GKF. All the regenerated films were cleaned with deionized water and dried at room temperature.

### 2.3. Measurements

#### 2.3.1. Sodium Dodecyl Sulfate-Polyacrylamide Gel Electrophoresis Analysis (SDS-PAGE)

Keratin’s molecular weight was tested by SDS gel electrophoresis using a DYCZ-24EN vertical electrophoresis instrument (Beijing Liuyi Biotechnology, Beijing, China). Firstly, the wool keratin solution was dialyzed using a cellulose dialysis bag to extract pure keratin. The bag filled with keratin solution was placed in ultrapure water for 48 h. The ultrapure water was changed every 3 h. After dialysis, the precipitated keratin was freeze-dried and made into keratin powder. Three μg keratin powder was added into 7 μL SDS loading buffer containing 5% *β*-mercaptoethanol, 10% SDS, and 50% glycerol. Keratin samples were denatured by boiling in the loading buffer for 5 min, and then 5 μL of this sample buffer solution was loaded into the lanes of the gels. Separation was performed at 80 V for 3 h. When the separation was finished, the gel was rinsed with ultrapure water and stained with Coomassie brilliant blue stain for 30 min. Destaining was done overnight in ultrapure water with gentle rotation and then the samples were compared with the protein molecular weight marker.

#### 2.3.2. Scanning Electron Microscopy (SEM)

The different keratin membrane samples were dried and sputter coated with gold and observed by Scanning Electron Microscope S4800 (Hitachi, Tokyo, Japan) at 5000–10,000× magnification and at an accelerating voltage of 10 kV.

#### 2.3.3. Fourier Transform Infrared Spectroscopy (FT-IR)

Fourier infrared scanning spectra of wool fibers and regeneration keratin samples were obtained using a Nicolet iS50 Fourier infrared spectrometer (Thermo Fisher Scientific, Waltham, MA, USA). Samples’ spectra were obtained in the wavenumber range of 500–4000 cm^−1^ and at a resolution of 4 cm^−1^. All the spectra were baseline corrected.

#### 2.3.4. Raman Spectra

All the spectra were recorded on a XploRA PLUS scanning imaging Raman spectrometer (Horiba, Kyoto, Japan). The laser beam on the sample was focused to a spot diameter of 1 μm under a 100× microscope objective. Spectra were recorded by scanning the 400–2000 cm^−1^ region, and the selected laser wavelength was 700 cm^−1^.

#### 2.3.5. X-ray Diffraction (XRD)

The crystal structure of regenerated keratin material was tested by D8 DISCOVER X-ray diffractometer (Bruker, Karlsruhe, Germany). Diffraction intensities were recorded with 2θ ranging from 5° to 40° at a scan speed of 0.05 s^−1^.

#### 2.3.6. Thermal Performance Analysis

The thermal properties and thermal degradation temperatures of wool fibers and regenerated keratin films were investigated by differential scanning calorimetry analysis (DSC) and thermogravimetric analysis (TG). 

The temperature of the samples was obtained by a DSC200F3 differential scanning calorimetric instrument (Netzsch, Bavaria, Germany). The samples were heated from 50 to 400 °C at a heating rate of 10 °C /min. The thermal degradation behavior of the samples was carried out by a STA449F3 thermogravimetry instrument (Netzsch, Bavaria, Germany). Keratin samples were tested using a ceramic crucible from 20 to 600 °C at a rate of 10 °C/min under nitrogen atmosphere.

#### 2.3.7. Tensile Strength Test

To evaluate the mechanical properties of the keratin films, the stress–strain curves were obtained with a 3369 tensile testing machine (Instron, Norwood, MA, USA). The keratin film samples, 50 mm in length and 2 mm in width, were tested at speed of 10 mm/min and with gauge length of 20 mm.

## 3. Results and Discussion

### 3.1. Electron Microscope Characterization of Keratin Films

**Figure 2:**
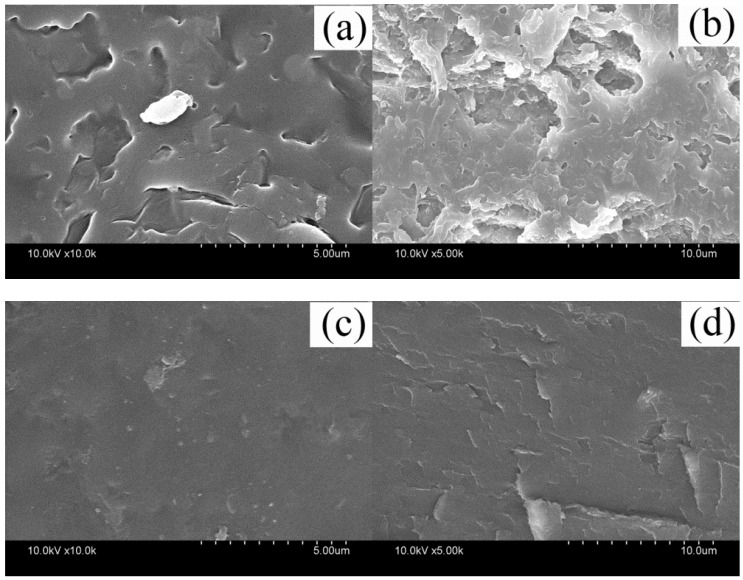
Scanning electron microscope images of (**a**) KF’s surface; (**b**) KF’s cross section; (**c**) GKF’s surface; (**d**) GKF’s cross section.

The SEM micrographs of regenerated keratin film (KF) and the graphene oxide-modified keratin film (GKF) are shown in Figure 2.

Figure 2a,b presents SEM images of KF’s surface morphology and cross-sectional morphology, respectively. The surface of the KF sample was very rough and porous, and there were also many cracks on the film. It also can be seen from Figure 2b that the internal structure of the film sample was also very loose. This disorderly and loosely arranged structure was a major cause of keratin film’s poor mechanical properties. However, when a certain amount of graphene oxide powder was evenly blended into keratin, the surface morphology of regenerated film significantly changed (Figure 2c,d). It can be seen from the images that the surface of GKF sample became smooth and the internal structure became uniform and compact. SEM test results illustrated that the physical structure of the regenerated keratin film was amended after the addition of graphene oxide. 

### 3.2. Keratin Films’ Molecular Weight Test

**Figure 3:**
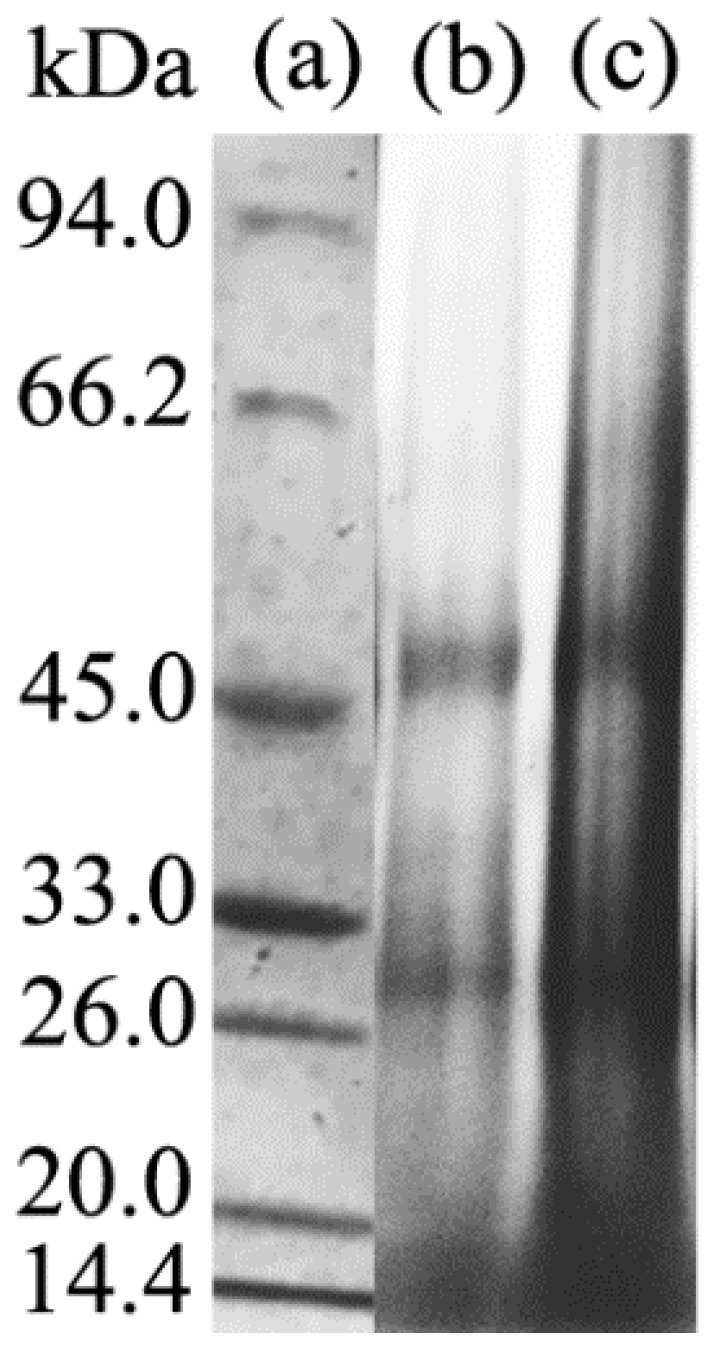
Gel electrophoresis test results. (**a**) protein molecular weight marker; (**b**) KF molecular weight distribution; (**c**) GKF molecular weight distribution.

SDS-PAGE gel electrophoresis analysis was carried out to characterize the molecular weight distribution of different keratin film samples and the test results are presented in Figure 3.

The protein macromolecules of the wool keratin films were mainly distributed in the range of 14.4 kDa, 26–33 kDa, and 45 kDa, and the molecular weight distribution of the KF and GKF samples were almost the same. This result indicated that the graphene oxide nanomaterial would not cause chemical cross-linkage between the protein macromolecular chains, so the change of the GKF sample’s morphology was not caused by the increased molecular weight of keratin.

### 3.3. Chemical Structure Change of Keratin

**Figure 4:**
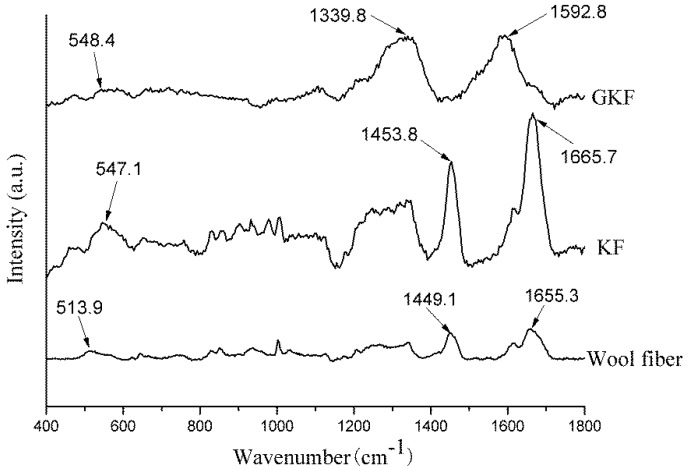
Raman spectra of wool fibers and regenerated keratin samples.

**Figure 5:**
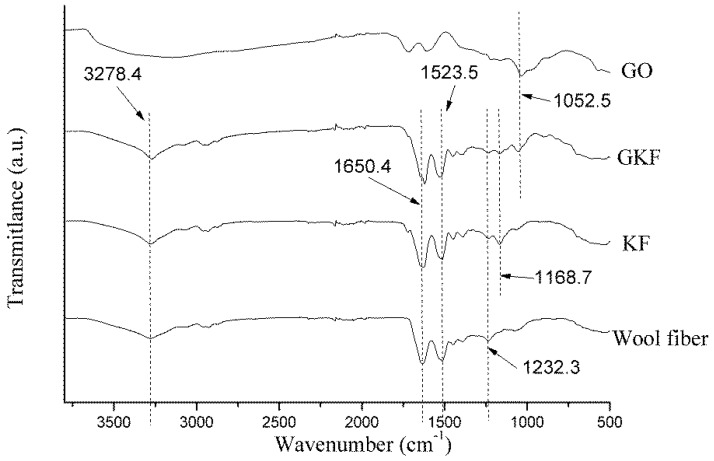
Fourier infrared spectra of wool fibers and regenerated keratin samples.

In order to investigate whether a chemical reaction occurs between keratin and graphene oxide, FT-IR and Raman analysis were used to analyze the chemical change of keratin. The test results are presented in Figure 4 and Figure 5.

The Raman spectra of wool fibers and regenerated keratin films are shown in Figure 4. According to the literature [25,26], disulfide bonds of different conformations form absorption peaks at different positions in Raman spectra. The disulfide bond of gauche-gauche conformation was located at 510 cm^−1^, gauche-trans at 520 cm^−1^, and trans-trans at 540 cm^−1^. It can be seen from Figure 4 that the absorption peak formed by the disulfide bonds in the wool fiber was located at 513.9 cm^−1^; however, the absorption peak in KF was blue-shifted (shift to higher frequencies) to 547.1 cm^−1^. This change indicated that during the dissolving and extracting process of regenerated keratin, the disulfide bonds in the fibers were disrupted and a random reconstruction occurred, leading to a transformation of disulfide bond conformation. Except for the change of the disulfide bonds, the Raman spectrum of the KF sample was generally the same as that of wool fibers, and the main Raman characteristic peaks of wool fibers were shown in Table 1 [27]. This phenomenon indicated that the regenerated keratin still retains the basic chemical properties of the protein.

The Raman spectrum of GKF changed significantly, compared with the wool fiber. The main absorption peaks appeared at 1339.8 cm^−1^ and 1592.8 cm^−1^, which represent the D and G bonds of graphene oxide, respectively [28]. Since the peak intensity of graphene oxide was large, the characteristic peaks of keratin were almost covered. Therefore, the Raman analysis results only proved that the GKF contained graphene oxide, but it cannot prove whether chemical reaction occurred between them.

Figure 5 shows the infrared spectra of the different samples. The spectra of wool fiber and regenerated keratin film were similar, and peaks mainly representing the characteristics of peptide bonds were located at 3278 cm^−1^, 1650 cm^−1^, 1523 cm^−1^, and 1232 cm^−1^, respectively. The characteristic peak appearing at 3278 cm^−1^ represented amide A, which was caused by stretching vibrations of the N–H bond in peptide chains [29]. The peak found at 1610–1680 cm^−1^ was associated with the stretching vibration of C=O (amide I). Many researchers [30,31] believe that the changes in the position of the amide I characteristic peak could exhibit different secondary structures of protein macromolecules. When the amide I peak is between 1650 and 1680 cm^−1^, the protein macromolecule mainly exhibits an α-helical conformation, and when it is located at 1610–1640 cm^−1^, the keratin was mainly in the *β*-sheet conformation. From the test results, it can be found that the amide I peak of wool fibers was located at 1650.5 cm^−1^, while the amide I of other two keratin films were located around 1625 cm^−1^, which indicated that after the wool fiber was dissolved and regenerated, the main arrangement of protein macromolecules was changed from the α-helical to *β*-sheet structure. The test samples’ amide II and amide III peaks were both located near 1523 cm^−1^ and 1232 cm^−1^, which represented the bending vibration of N–H bonds and stretching vibration of C–N bonds, respectively. The positions of each sample’s main characteristic peaks are presented in Table 2.

Compared with the spectrum of wool fibers, the KF and GKF spectra showed a new peak at 1168 cm^−1^ caused by S–O bonds, which are not present in the wool. This proved that the disulfide bonds in wool were disrupted during the process of dissolution and regeneration, and parts of the disulfide bond were oxidized to form chemical groups with S–O bonds. The reaction is represented in Formula (1), wherein the disulfide bonds of wool are oxidized to form sulfonate.

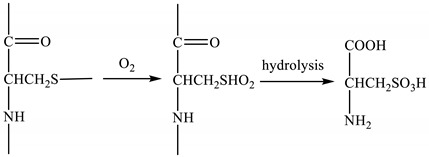
(1)

A new absorption peak (O–C–O) appeared at 1052 cm^−1^ in the GKF spectrum, indicating that graphene oxide was added into the film sample [28]. At the same time, the main peaks of the GKF sample did not change significantly compared with wool fibers, and no other new absorption peaks appeared in the GKF spectrum. This result indicated that graphene oxide did not chemically react with wool keratin, and they mainly interacted through hydrogen bonds and Van der Waals forces.

### 3.4. Effect of Graphene Oxide on the Crystal Structure of Regenerated Keratin

**Figure 6:**
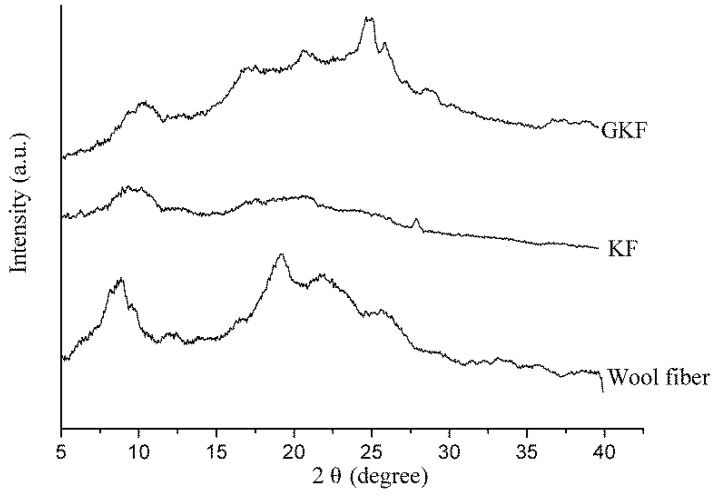
X-ray diffraction analysis of wool fibers and regenerated keratin samples.

**Figure 7:**
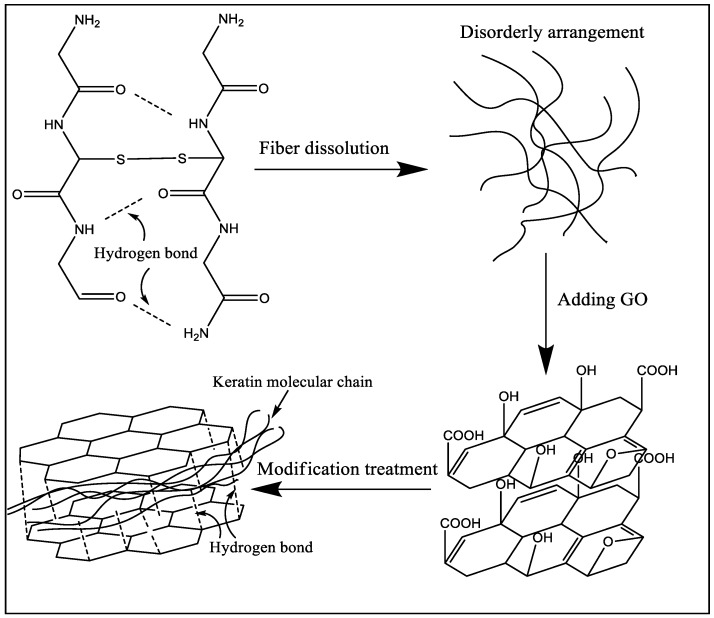
The mechanism of graphene oxide modification of keratin.

XRD analysis can be used to analyze the changes of different keratin materials’ crystal structures. The test results of wool fibers and keratin films are presented in Figure 6. According to information reported in previous literature [32,33,34], there were three distinct diffraction peaks in the XRD test results for wool keratin. The first peak that appeared near 10° in the XRD plot was an overlapping peak of α-helix structure and *β*-sheet structure. The peaks at 19° and 22° were attributed to the α-helix characteristic peak and *β*-sheet characteristic peak, respectively. The test results of Figure 6 showed that the macromolecular arrangement of regenerated keratin films was greatly changed compared to that of wool fibers. The X-ray diffraction result of wool fibers was basically consistent with the results mentioned in the literature, which indicated that the main arrangements of keratin macromolecules in fiber were α-helix structure, *β*-sheet structure, and amorphous arrangement. When wool fibers were dissolved and regenerated to form keratin film (KF), the diffraction peaks corresponding to crystal structure (α-helix structure and *β*-sheet structure) were remarkably reduced, and the proportion of amorphous region was increased. It was obviously indicated that the secondary structure of part of the keratin molecular chain was destroyed during the fiber dissolution process [35]. These molecular chains were disorderedly entangled together and eventually formed the amorphous region in the KF sample. For GKF, the sheet structure and interaction force of GO had orientation and constraining effects on the arrangement of keratin macromolecules, which prompted keratin molecular chains to be more ordered, such that the crystallinity of GKF was significantly improved compared to the KF sample. The effect of graphene oxide on keratin is shown in Figure 7. As can be seen from the schematic, during the dissolution process, the chemical bonds between wool keratin structures were disrupted and these proteins formed macromolecular chain segments of varying lengths. These molecular chains were randomly distributed and aggregated in solution, so that more amorphous regions were generated during the formation of regenerated film. After graphene oxide was added into the keratin solution, the macromolecular protein chains were arranged in a parallel or folded form between the graphene oxide sheets, and this change caused the regenerated film to have a more crystalline structure (*β*-sheet structure). The XRD test result was consistent with the SEM result, which proved that GO can significantly enhance the crystallization degree and improve the physical properties of regenerated keratin materials.

### 3.5. Thermal Performance Analysis

**Figure 8:**
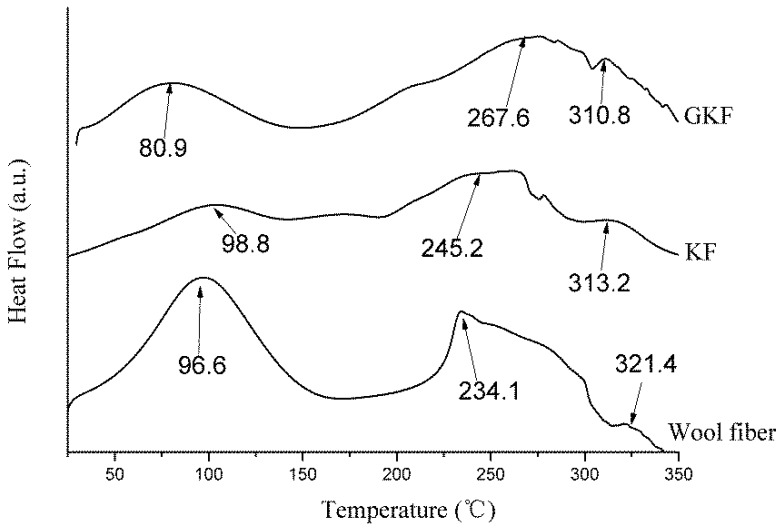
Differential scanning calorimetry (DSC) thermograms of wool fibers and regenerated keratin films.

**Figure 9:**
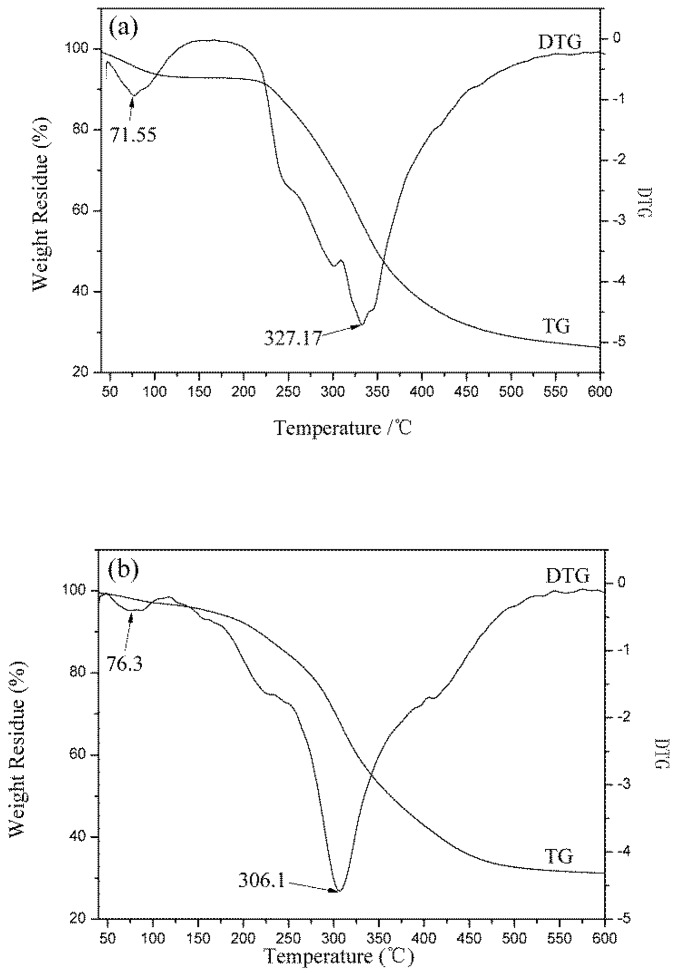

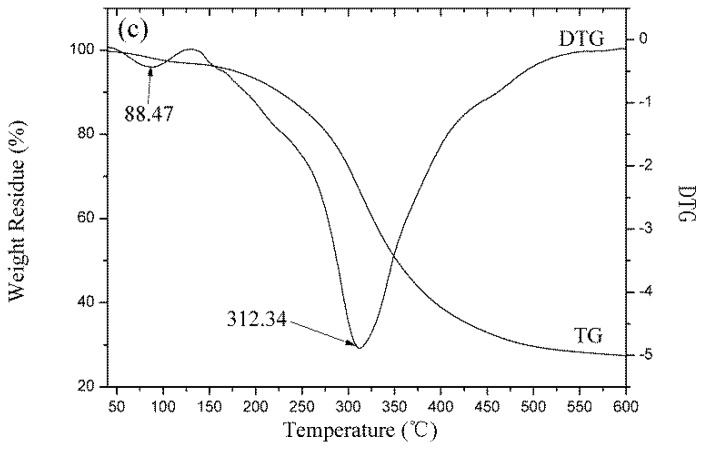
Thermogravimetric trace of (**a**) wool fibers; (**b**) KF; (**c**) GKF.

The change of molecular structure will affect the materials’ thermal properties. The thermal properties of wool fiber and keratin films were studied by DSC and TG analysis. The test results are presented in Figure 8 and Figure 9.

It can be seen in Figure 8 that the endotherm curves of wool fibers and regenerated keratin materials were similar. Three endothermic peaks appeared in the temperature range from 25 °C to 350 °C. The first two peaks were more obvious and corresponded to the changes of the keratin material’s molecular structure during the heating process [36,37]. Firstly, an endothermic peak appeared near 90 °C on the DSC curves of the test samples which was caused by the evaporation of water in the material. The second endothermic peak was mainly caused by high temperature destruction of the secondary structure between protein macromolecules. These peaks occurred in the 230–260 °C temperature range and the endothermic temperature of the wool fibers was lower than that of the regenerated keratin films. This phenomenon was due to the presence of more α-helical structure in the crystalline region of the wool fiber, whereas the macromolecular arrangement of the regenerated keratin film was dominated by the *β*-sheet amorphous structures. Since the interaction between macromolecular protein chains in *β*-sheet structures is stronger than that of α-helical structures, the macromolecular secondary structure in the regenerated keratin film needed to absorb more heat when it was thermally decomposed. At the same time, the DSC curve of wool fibers had a much larger absorption peak area than the regenerated protein material, which indicates that there are more crystalline regions in the fiber. The third peak at 300–320 °C was characteristic of the thermal degradation of the macromolecular protein chains. Because the macromolecular protein chains were disrupted partially during the dissolution and regeneration processes, the temperatures required for thermal degradation of macromolecules in the regenerated keratin films (310.8 °C and 313.1 °C) were lower than that of wool fibers (321.4 °C).

The thermogravimetric curves of fibers and keratin film samples are shown in Figure 9. The results indicated that as the temperature increased, the protein material gradually degraded and lost weight. The thermal degradation process of keratin materials mainly consisted of two stages [3]: the weight loss in the first stage was caused by the evaporation of water from the samples. Wool fiber has good moisture absorbency and it contains many hydrophilic groups. Thus, wool fibers’ weight loss ratio at this stage was high (6.20%), while the weight loss ratio of the KF and the GKF samples were low (2.48% and 3.01%, respectively). The second stage was the thermal degradation process. From the TG-DTG test results, it can be found that the fastest thermal degradation temperature of wool fiber was 327.17 °C, which was the highest among the samples. The fastest thermal degradation temperature of the KF sample was 306.1 °C, and for GKF it was 312.34 °C, which are not significantly different. This result indicated that the thermal stability of the regenerated keratin films were weakened compared to wool fibers due to the changes of protein structure and the decrease of molecular weight. At the same time, the graphene oxide material only affected the aggregation structure of the macromolecules without changing the structure and properties of the keratin. Thus, the thermal degradation temperature (macromolecule chain rupture temperature) of the regenerated keratin film after modification with GO did not show significant improvement.

### 3.6. Mechanical Properties of Regenerated Keratin Films

**Figure 10:**
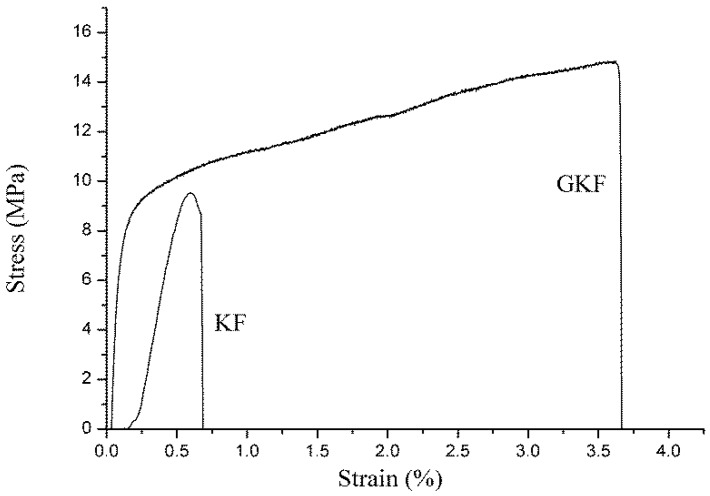
Tensile mechanical properties of different keratin film samples.

The stress–strain curves of regenerated keratin films are shown in Figure 10.

Mechanical properties are important indicators for examining whether materials can be used in actual applications. Unmodified regenerated keratin materials are often hard and brittle due to their structure and properties, so their mechanical properties are extremely poor. The modification of keratin with GO was intended to improve the mechanical properties of the regenerated protein materials, and then to use the modified regenerated keratin for the preparation of protein fibers. The test results showed that the tensile strength of the regenerated keratin film was significantly improved after the addition of graphene oxide. The unmodified keratin film (KF) was hard and brittle, and it was prone to breakage during testing. Its breaking strain was only 0.67%. The reason for this phenomenon is that the macromolecules were disordered during the regeneration process, and these molecular chains formed a cross-linked network structure, which made the material rigid. Incorporation of GO resulted in a significant increase of the GKF sample’s strength. The addition of graphene oxide promoted the ordered arrangement of the molecular chains, which helped to improve the crystallinity of the keratin film. Under the same test conditions, the maximum tensile stress of GKF can reach 14.7 MPa, which is almost double that of the KF sample. The tensile strain was also increased to 3.65%. This result proved that GO could greatly improve the mechanical properties of regenerated keratin material. The breaking strength of the GKF sample was 1.5 times higher than that of the KF sample, and the breaking elongation of the keratin film was increased 5.4-fold, making the keratin material more suitable for preparation of the regenerated protein fibers.

## 4. Conclusions

In this work, graphene oxide (GO) was used to modify regenerated keratin film to improve its mechanical properties. The modification effect and reaction mechanism of GO on keratin were investigated in detail. Through the SEM analysis of different regenerated film samples, it was found that the addition of GO can effectively improve the physical morphology of keratin film and make the material’s structure more uniform. The modification mechanism of GO on keratin was analyzed by SDS-PAGE, Raman spectroscopy, Fourier infrared spectroscopy, and XRD tests. The test results showed that there is no obvious chemical reaction between the GO and keratin macromolecules, and that they mainly interacted with each other via hydrogen bonding and van der Waals forces. Therefore, after modification by GO, the regenerated materials retained the inherent chemical properties of wool keratin, but their physical structure was changed. The XRD test results demonstrated that GO can promote the conversion of keratin macromolecules from a disordered distribution to a folded or parallel arrangement, which improved the crystallization degree of the material. Thus, this modification method can effectively improve the structure and properties of regenerated keratin film. In addition, the thermal stability and mechanical properties of GO-modified keratin films were significantly enhanced compared to unmodified keratin. Therefore, further research on the modification process of keratin with graphene oxide will help researchers to obtain regenerated keratin polymer materials with excellent performance in the future.

## Figures and Tables

**Table 1:** Raman characteristic peak distribution of the test samples (cm^−^^1^).

Wool Fiber	KF	Assignment
1655.3	1665.7	Amide I
1613.5	1611.9	Tyrosine
1449.1	1453.8	CH_2_ bending mode
1338.5	1339.9	Tryptophan
1248.2	1248.1	Amide III
1002.3	1002.9	Phenylalanine
931.1	931.9	C–C stretch
663.2	666.8	C–S stretch
513.9	547.1	S–S

**Table 2:** Characteristic peak distribution of the test samples (cm^−^^1^).

Samples	Amide A	C–H	Amide I	Amide II	Amide III	S–O	C–O
wool fibers	3278.4	2947.2	1650.4	1523.5	1232.3		
KF	3271.2	2943.3	1627.6	1524.4	1238.5	1168.7	
GKF	3268.8	2945.2	1620.4	1524.9	1234.7	1169.1	1052.5
GO							1045.2
